# Supplementary material for: Atomically dispersed asymmetric cobalt electrocatalyst for efficient hydrogen peroxide production in neutral media
Source: Nat Commun. 2024 May 14;15:4079. doi: 10.1038/s41467-024-48209-0 (PMC11093996; doi:10.1038/s41467-024-48209-0)
Supplement: Supplementary file 1 — Supplementary Information [file 41467_2024_48209_MOESM1_ESM.pdf]

Supporting Information

Atomically Dispersed Asymmetric Cobalt Electrocatalyst for Efficient Hydrogen Peroxide Production in Neutral Media

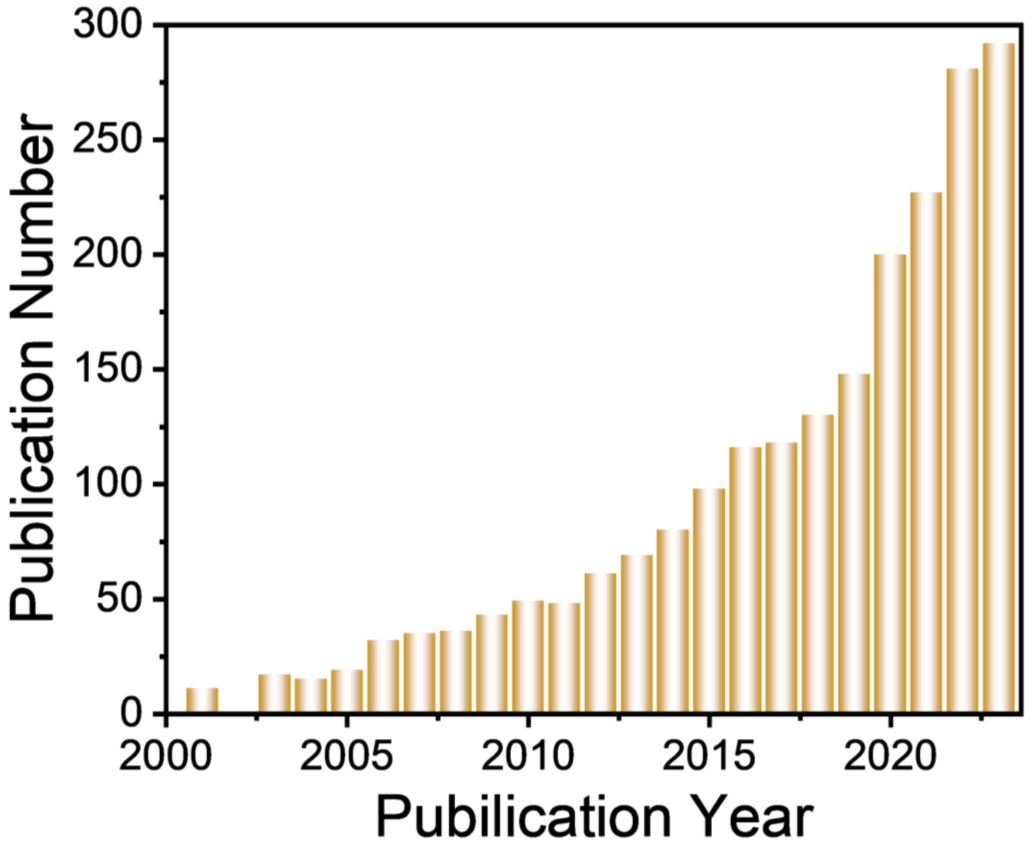

Figure S1. Publication number per year between 2000-2023 regarding the topic of 'electrochemical or electrocatalytic', 'oxygen reduction reaction', and 'hydrogen peroxide'. The data was collected from the *web of science* website (https://www.webofscience.com/wos/woscc/basic-search) on 05th February 2024

The literature analysis in Figure 1b (in the manuscript) is based on the data from recent 4 years (2020-2023). 859 publications were analyzed except review papers. Moreover, some topics in these 859 publications such as H_2O_2 sensors, photocatalysts, CO_2 reduction, nitrogen reduction reaction, and $4e^-$ ORR fuel cells, unclassified reviews, etc. were ruled out manually. Therefore, 587 publications in total were analyzed finally, and the classification methods are listed below.

Table S1. Classification method of $2e^-$ ORR catalysts.

Third	Second	**First**
Defects	Carbon (59)	**Carbon (59)**
Porous structures		
Hydrophilicity		
Gas diffusion electrode		
Carbon felt		
Others		
O-doped	Metal-free heteroatom doped carbon (150)	**Heteroatom doped carbon (331)**
N-doped		
B-doped		
F-doped		
S-doped		
COFs		
Others		
Metal-N-doped	Non-noble metal heteroatom carbon (156)	
Metal-C/O/P/S-doped		
Metal oxide-doped		
Metal hydroxide-doped		
Others		
Metal nanoparticle	Noble metal heteroatom carbon (25)	
Metal-N-doped		
Metal Cluster		
Others		
Metal oxide	Non-noble metal (82)	**Metal (106)**
Perovskite		
Layered double hydroxide		
Metal hydroxide		
MXenes		
Others		
Metal oxide	Noble metal (24)	
Metal P/Se/S		
Metal alloy		
Others		
Computational Study	Others (65)	**Others (65)**
Quantifying method		
Non-aqueous media		

Table S2. Classification method of pH conditions.

Media	pH	Catalysts
0.1 M $HClO_4$	Acidic	Carbon (18)
		Metal-free heteroatom doped carbon (51)
0.05 M H_2SO_4		Non-noble metal heteroatom carbon (70)
0.5 M H_2SO_4		Noble metal heteroatom carbon (17)
0.05 M Na_2SO_4 (pH=3)		Non-noble metal (17)
		Noble metal (15)
Others		Others (12)
0.1 M PBS	Neutral	Carbon (31)
0.05 M Na_2SO_4		Metal-free heteroatom doped carbon (41)
0.1 M Na_2SO_4		
0.1 M $NaClO_4$		Non-noble metal heteroatom carbon (41)
0.5 M NaCl		
1 M K_2SO_4		Noble metal heteroatom carbon (1)
1 M Na_2SO_4		Non-noble metal (16)
1 M Na_2CO_3		Noble metal (6)
Others		Others (13)
0.1 M KOH	Alkaline	Carbon (29)
		Metal-free heteroatom doped carbon (99)
0.1 M NaOH		Non-noble metal heteroatom carbon (90)
0.05 M Na_2SO_4 (pH=13)		Noble metal heteroatom carbon (10)
		Non-noble metal (62)
Others		Noble metal (7)
		Others (8)
$[nBu_4][PF_6]$	Non-aqueous	Others (2)

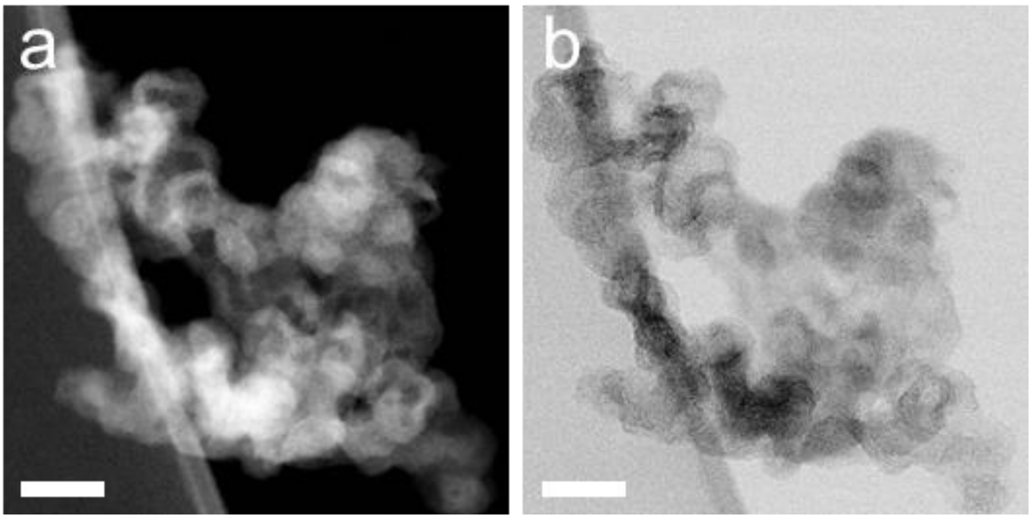

Figure S2. STEM images of CoNCB. (a) HAADF-STEM image and (b) ABF-STEM image of CoNCB. The magnification is 60k and scale bar is 50 nm.

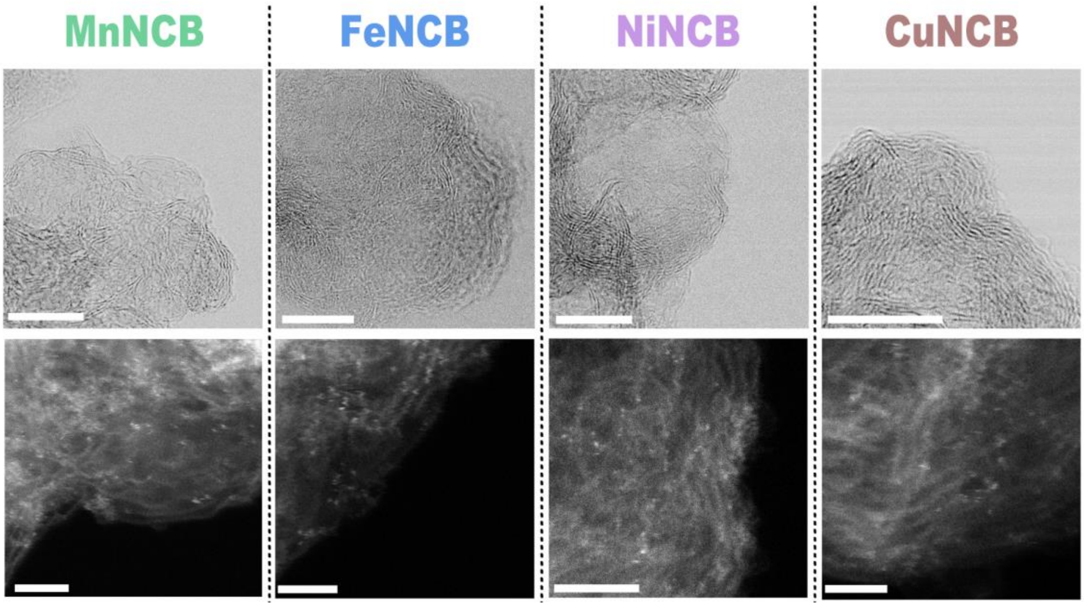

Figure S3. ABF- and ADF-STEM images of MnNCB, FeNCB, NiNCB, and CuNCB materials. Scale bar of top images: 10 nm, bottom images: 2 nm.

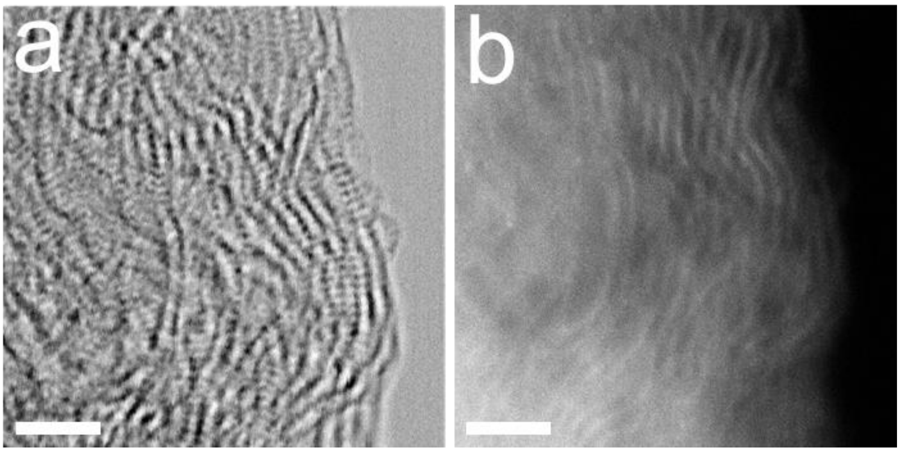

Figure S4. STEM images of NCB. (a) ABF- and (b) HAADF- STEM images of metal-free NCB material. Scale bar of both: 2 nm.

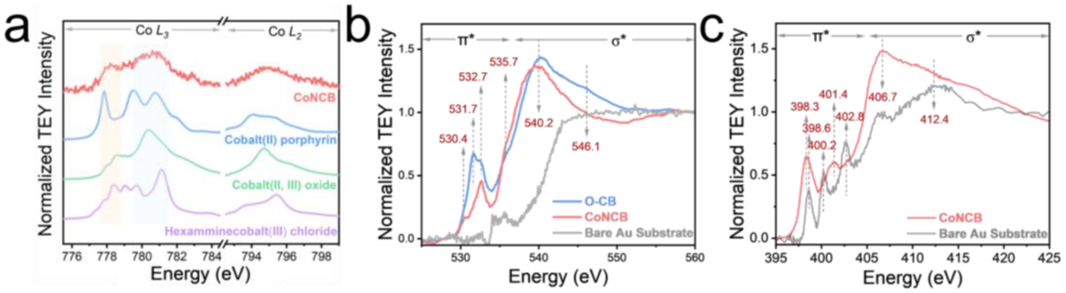

Figure S5. NEXAFS spectra. (a) Co L-edge NEXAFS spectra. Spectra are displaced vertically for clarity. (b) O K-edge NEXAFS spectra. (c) N K-edge NEXAFS spectra.

Supplementary Note 1. The identification of N K-edge features was struggling due to the contamination from the beamline setup. The contamination was not supposed to be present on the clean bare Au substrate. Silicon window was used to measure this sample at B07-B beamline at Diamond Light Source, to avoid the N K-edge interference from silicon nitride window. The contamination was probably due to the glue used for the silicon window assembly after discussion with beamline scientists. We apologize for this as we cannot find a good way to obtain the real N K-edge at the moment. This supplementary note is to prevent causing misleading understanding.

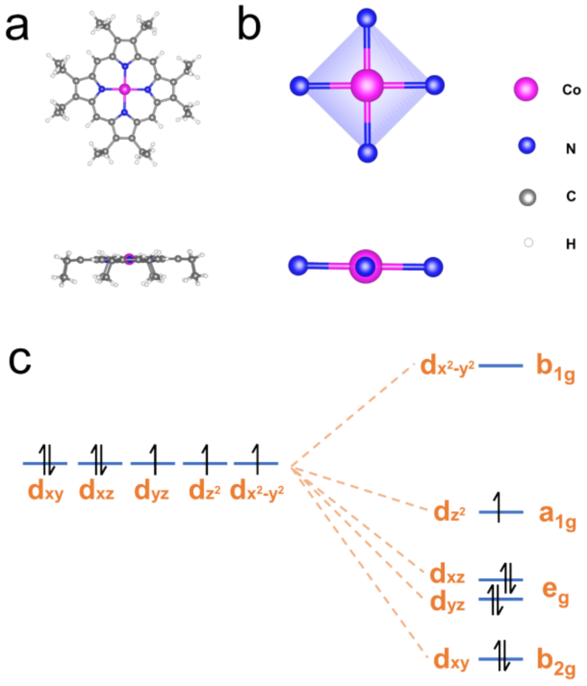

Figure S6. Schematic drawings of CoPr structure. (a) front view and top view. (b) Simplified illustration of D_{4h} symmetry. (c) Schematic illustration of 3d orbital splitting of low spin CoPr with D_{4h} symmetry.

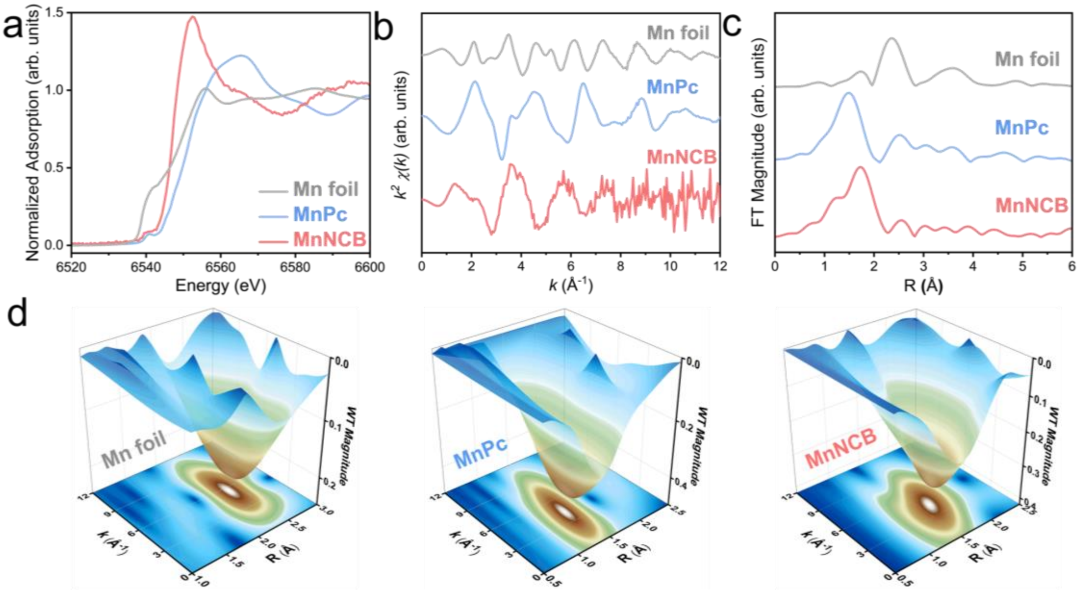

Figure S7. Mn K-edge XAFS spectra. (a) Mn K-edge XANES spectra of Mn foil, MnPc, and MnNCB. (b) k^2-weighted extracted EXAFS signals. Spectra are displaced vertically for clarity. (c) FT magnitudes of k^2-weighted EXAFS spectra without phase correction. Spectra are displaced vertically for clarity. (d) k^2-weighted WT-EXAFS 3D contour plots of Mn foil, MnPc, and MnNCB.

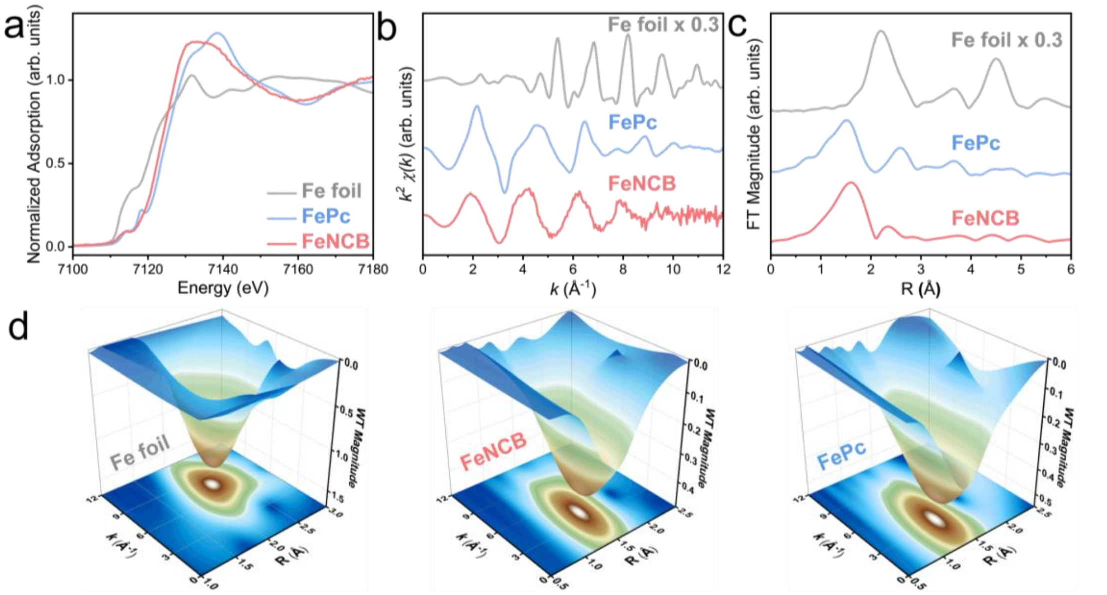

Figure S8. Fe K-edge XAFS spectra. (a) Fe K-edge XANES spectra of Fe foil, FePc, and FeNCB. (b) k^2-weighted extracted EXAFS signals. Spectra are displaced vertically for clarity. (c) FT magnitudes of k^2-weighted EXAFS spectra without phase correction. Spectra are displaced vertically for clarity. (d) k^2-weighted WT-EXAFS 3D contour plots.

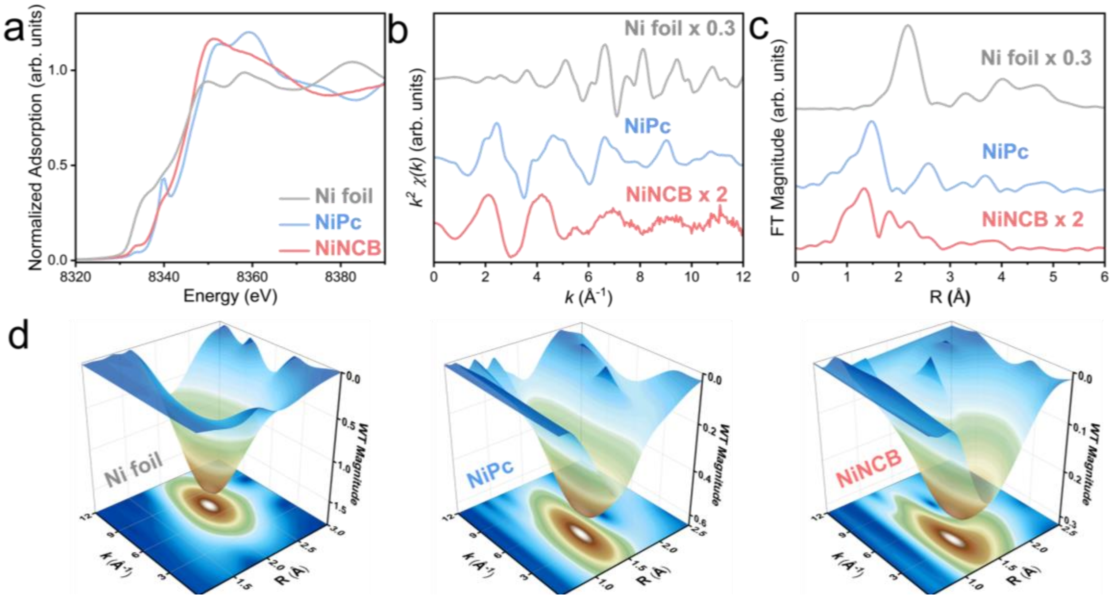

Figure S9. Ni K-edge XAFS spectra. (a) Ni K-edge XANES spectra of Ni foil, NiPc, and NiNCB. (b) k^2-weighted extracted EXAFS signals. Spectra are displaced vertically for clarity. (c) FT magnitudes of k^2-weighted EXAFS spectra without phase correction. Spectra are displaced vertically for clarity. (d) k^2-weighted WT-EXAFS 3D contour plots of Ni foil, NiPc, and NiNCB.

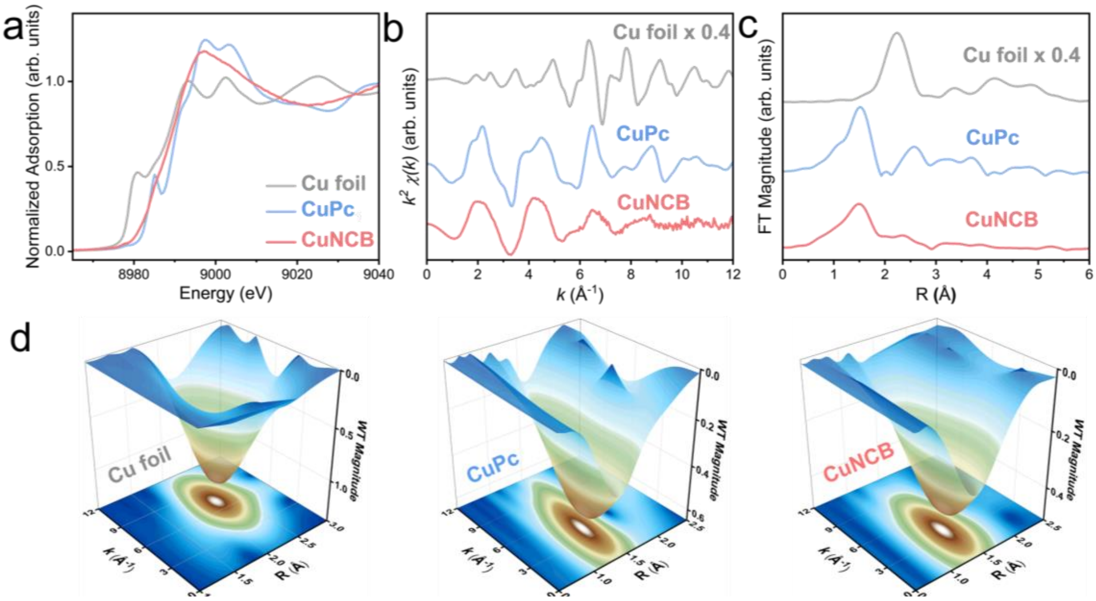

Figure S10. Cu K-edge XAFS spectra. (a) Cu K-edge XANES spectra of Cu foil, CuPc, and CuNCB. (b) k^2-weighted extracted EXAFS signals. Spectra are displaced vertically for clarity. (c) FT magnitudes of k^2-weighted EXAFS spectra without phase correction. Spectra are displaced vertically for clarity. (d) k^2-weighted WT-EXAFS 3D contour plots of Cu foil, CuPc, and CuNCB.

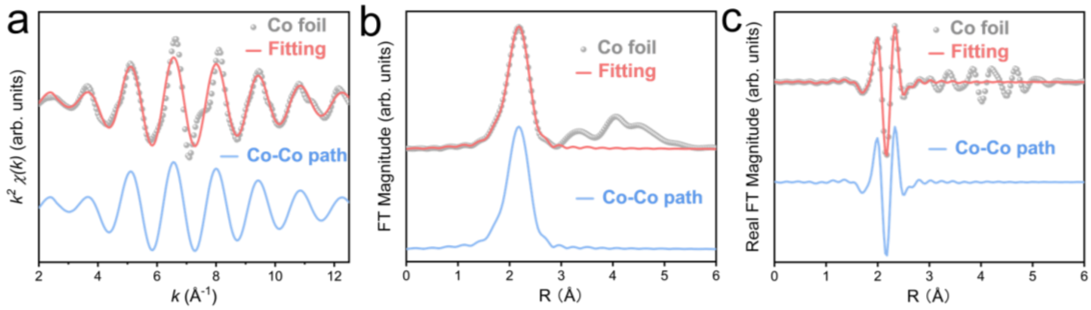

Figure S11. EXAFS fitting of Co foil. (a) k^2-weighted extracted EXAFS signal of experimental result of Co foil and related fitting result using Co-Co path. (b) FT magnitudes of k^2-weighted EXAFS spectra without phase correction. (c) Real part of FT magnitudes.

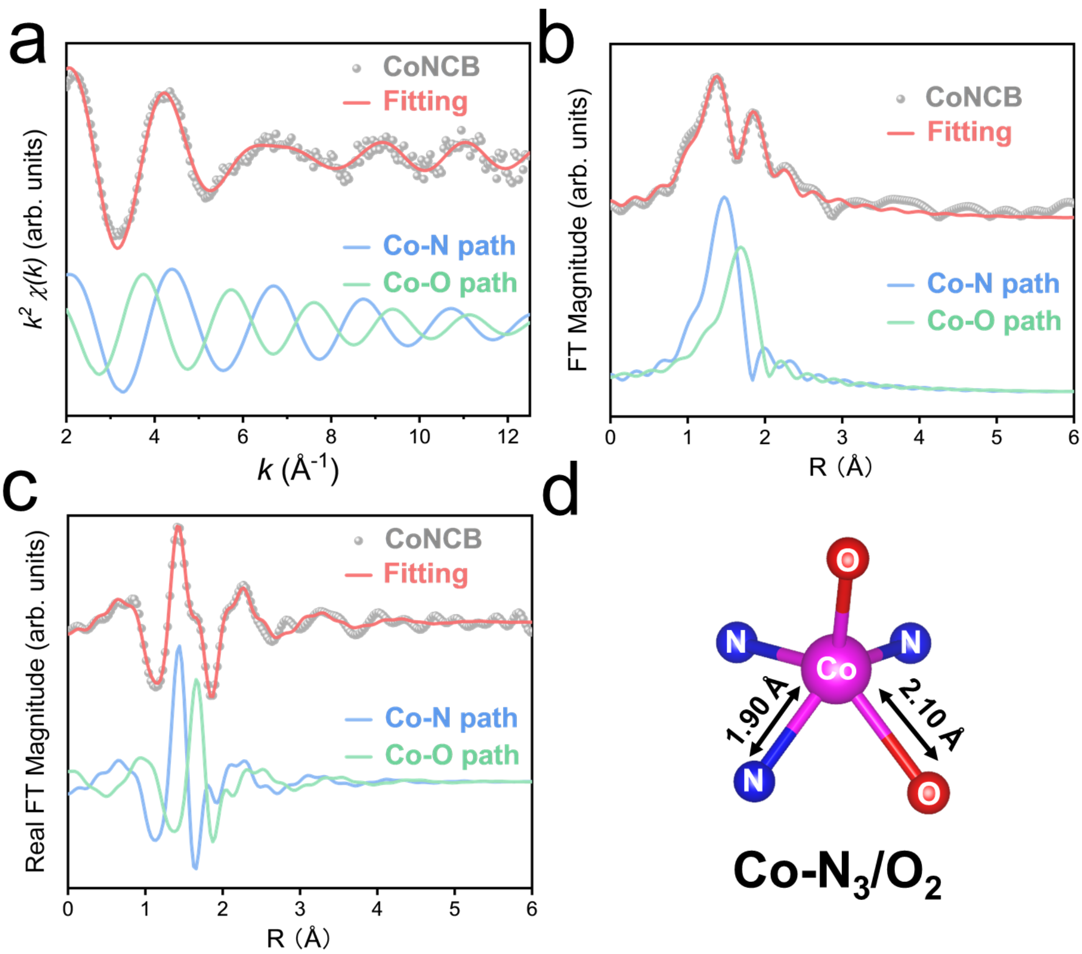

Figure S12. EXAFS fitting of CoNCB. (a) k^2-weighted extracted EXAFS signal of experimental result of CoNCB and related fitting result using Co-N path and Co-O path. (b) FT magnitudes of k^2-weighted EXAFS spectra without phase correction. (c) Real part of FT magnitudes. (d) Fitted structure model.

.

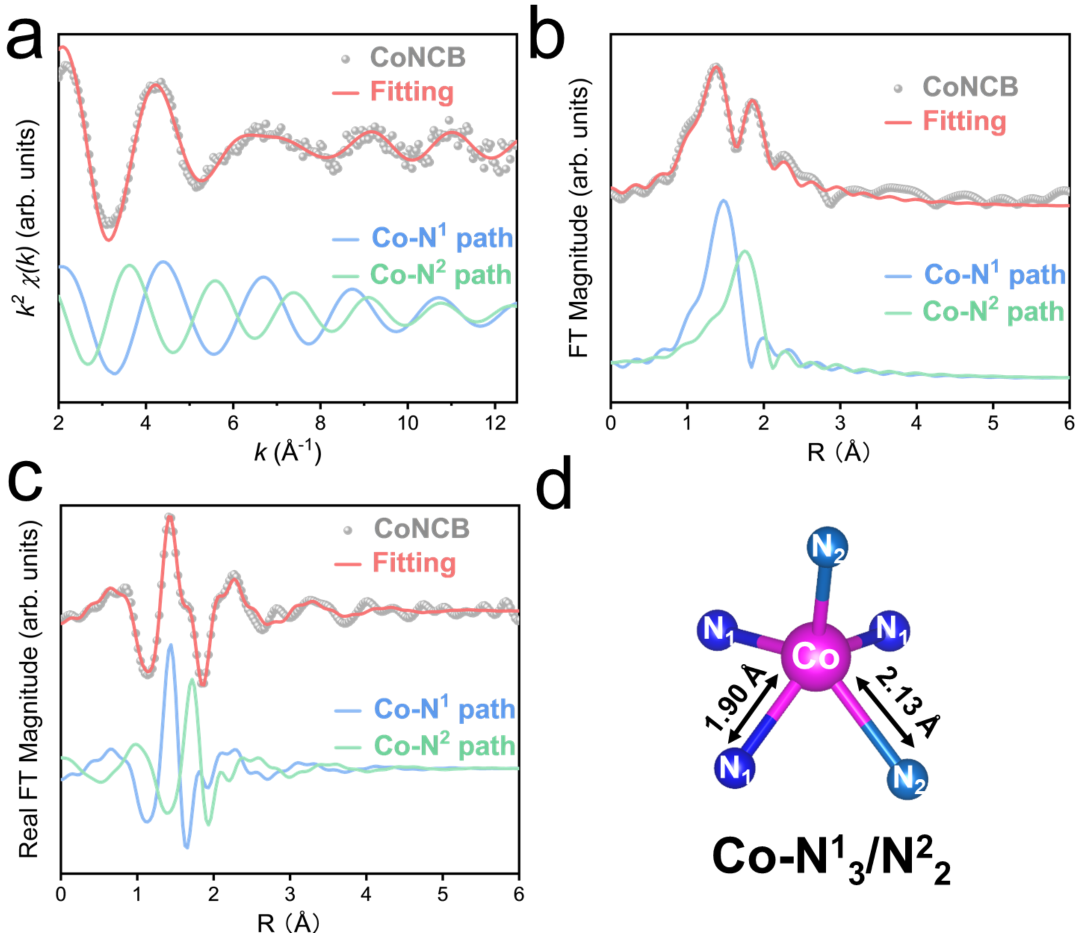

Figure S13. EXAFS fitting of CoNCB. (a) k^2-weighted extracted EXAFS signal of experimental result of CoNCB and related fitting result using two Co-N paths. (b) FT magnitudes of k^2-weighted EXAFS spectra without phase correction. (c) Real part of FT magnitudes. (d) Fitted structure model.

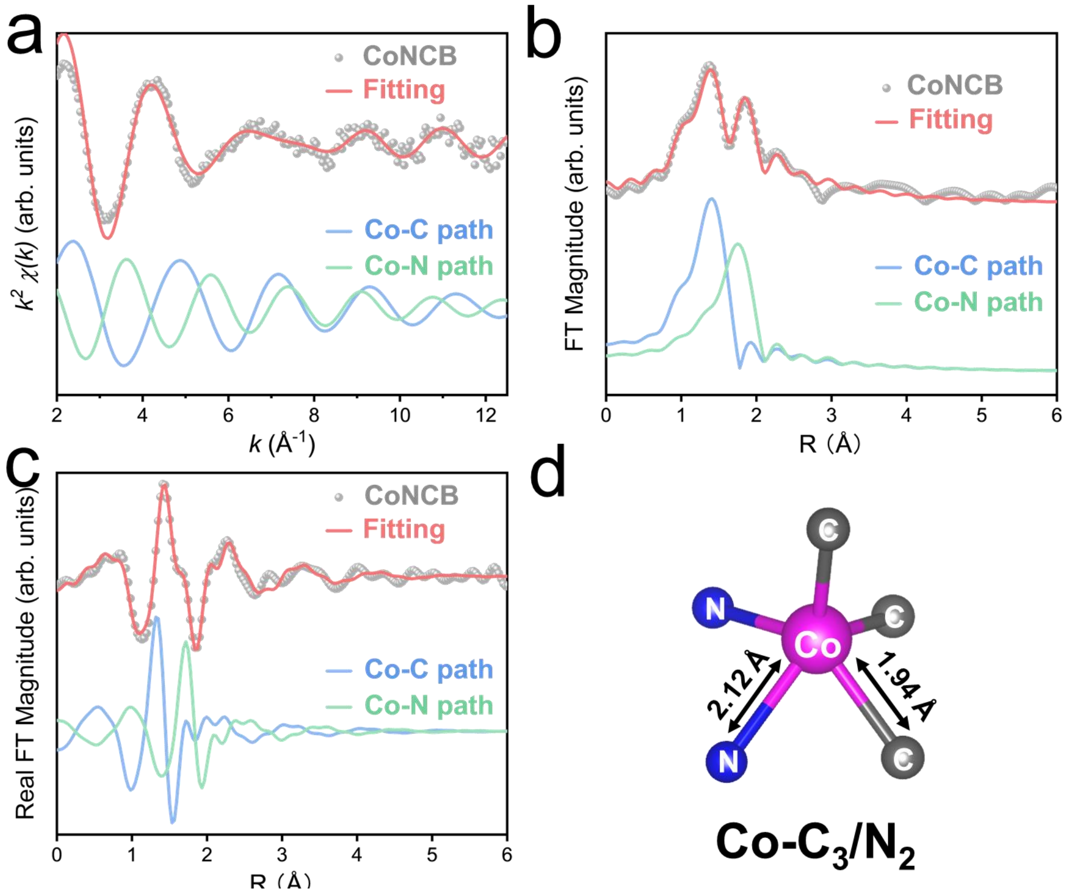

Figure S14. EXAFS fitting of CoNCB. (a) k^2-weighted extracted EXAFS signal of experimental result of CoNCB and related fitting result using Co-C path and Co-N path. (b) FT magnitudes of k^2-weighted EXAFS spectra without phase correction. (c) Real part of FT magnitudes. (d) Fitted structure model.

Table S3. Fitting parameters of Co-K edge EXAFS of Co foil.*

Sample	Path	S_0^2	R (Å)	C.N.	σ^2 ($x10^{-3}$Å^2)	ΔE_0	R factor (%)
Co foil	Co-Co	0.74±0.02	2.49±0.00	12^b	6.20±0.26	7.2±0.4	0.17

* S_0^2 is the amplitude reduction factor; R is the interatomic distance (the distance between absorber and backscatter atoms) in the fitting results; C.N. is the coordination number; σ^2 is Debye-Waller factor (a measure of thermal and static disorder in adsober-scatterer distances); ΔE_0 is the edge-energy shift; R factor is used to evaluate the goodness of the fitting results.

[b] This value is fixed to determine the S_0^2.

Fitting range: $3.25 \le k$ (Å^{-1}) ≤ 14.25; $1.3 \le$ R (Å) ≤ 2.9.

Table S4. Fitting parameters of Co-K edge EXAFS of CoNCB.*

	Path	R (Å)	C.N.	σ^2 ($x10^{-3}$ Å^2)	ΔE_0	R factor (%)
1	Co-N	1.90±0.01	3.0±0.3	4.5±1.1	3.1±1.2	0.5
	Co-O	2.10±0.01	2.1±0.2	4.5±1.1	3.1±1.2	
2	Co-N^1	1.90±0.01	2.8±0.4	4.6±1.3	3.3±1.5	0.7
	Co-N^2	2.13±0.01	2.3±0.2	4.6±1.3	3.3±1.5	
3	Co-C	1.94±0.02	2.9±0.6	3.3±2.0	3.7±2.0	1.9
	Co-N	2.12±0.02	2.4±0.4	3.3±2.0	3.7±2.0	

* The σ^2 value is set to be the same for each path in the fitting process. The ΔE_0 value is set to be the same for each path in the fitting process. S_0^2 was fixed to be 0.74 determined by Co foil fitting.

Fitting range: $3.1 \leq k$ (Å^{-1}) ≤ 12.3; $1 \leq R$ (Å) ≤ 2.6.

Due to the limit of independent points, only the combination of two paths was considered here. All the attempts showed well-matched fitting results, demonstrating the infeasibility of EXAFS fitting in identifying the accurate coordination information of C/N/O. Moreover, the ± 20% error bounds in the fitting parameters make it more challenging to identify the accurate coordination number and distance of cobalt species in CoNCB based on the EXAFS analysis. To the best of our knowledge, the same issues exhibit in all the metal-C/N/O materials prepared by the annealing method and without known electronic structure, and there is not a feasible way to solve this issue currently for the metal-C/N/O materials with very low loading of metal. However, this issue has been consciously ignored by previous research in which many ambiguous or even misleading conclusions were reported.

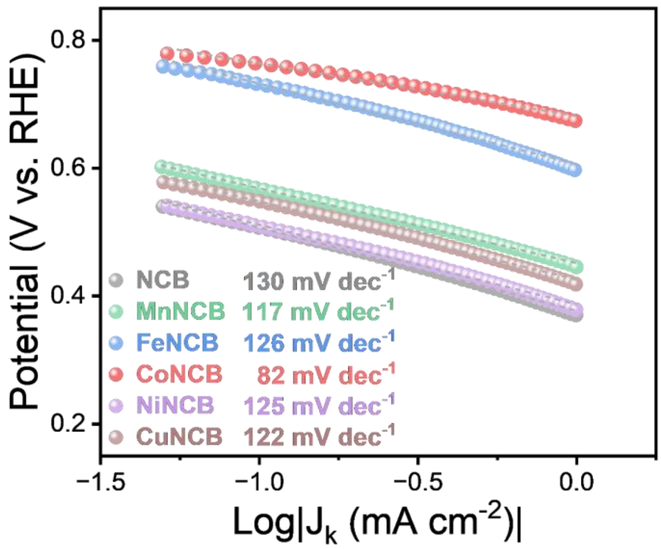

Figure S15. Calculated Tafel plots from disk LSV current density; labels show the corresponding Tafel slopes. Koutecky-Levich equation was used with 5.8 mA cm^{-2} as the theoretical limiting current of $4e^-$ ORR process.

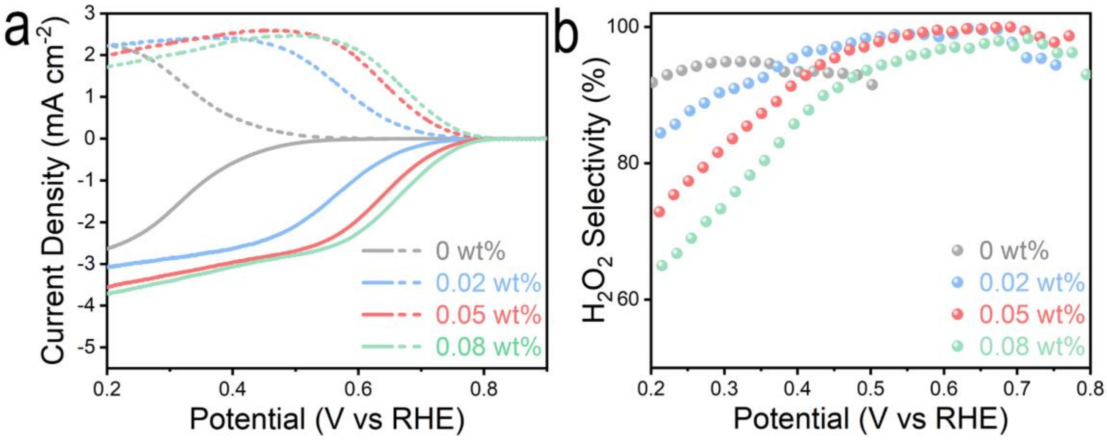

Figure S16. Effect of Co loading. (a) ORR polarization curves of disk current density (solid line) and ring current density (dash line) of CoNCB with different Co loading content in 0.1 M PBS (pH = 7). (b) Calculated H_2O_2 selectivity (H_2O_2 %). The optimal loading content is determined to be 0.05 wt % with both high activity and selectivity.

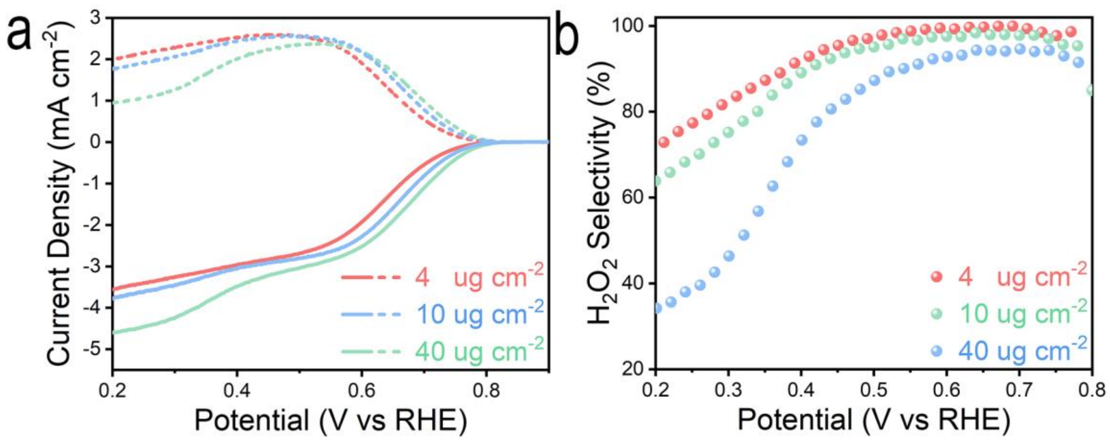

Figure S17. Effect of catalyst loading. (a) ORR polarization curves of disk current density (solid line) and ring current density (dash line) of CoNCB with different catalyst loading in 0.1 M PBS (pH = 7). (b) Calculated H_2O_2 selectivity (H_2O_2 %). They exhibited similar ring current density in the potential range of 0.55-0.75 eV, but the H_2O_2 selectivity significantly decreased for catalyst loading of 40 ug/cm^2, indicating that the generated peroxide was trapped within catalyst layer and further reduced to H_2O. Therefore, we opted for a very thin layer of catalyst (4 ug/cm^2).

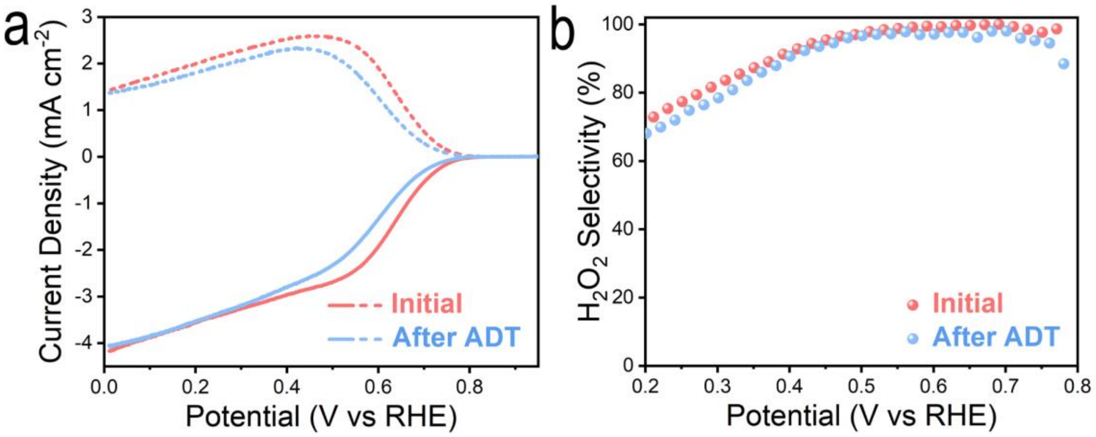

Figure S18. Durability test. (a) ORR polarization curves of disk current density (solid line) and ring current density (dash line) of initial CoNCB and after 5,000 cycles of CV in 0.1 M PBS (pH = 7). (b) Calculated H_2O_2 selectivity (H_2O_2 %).

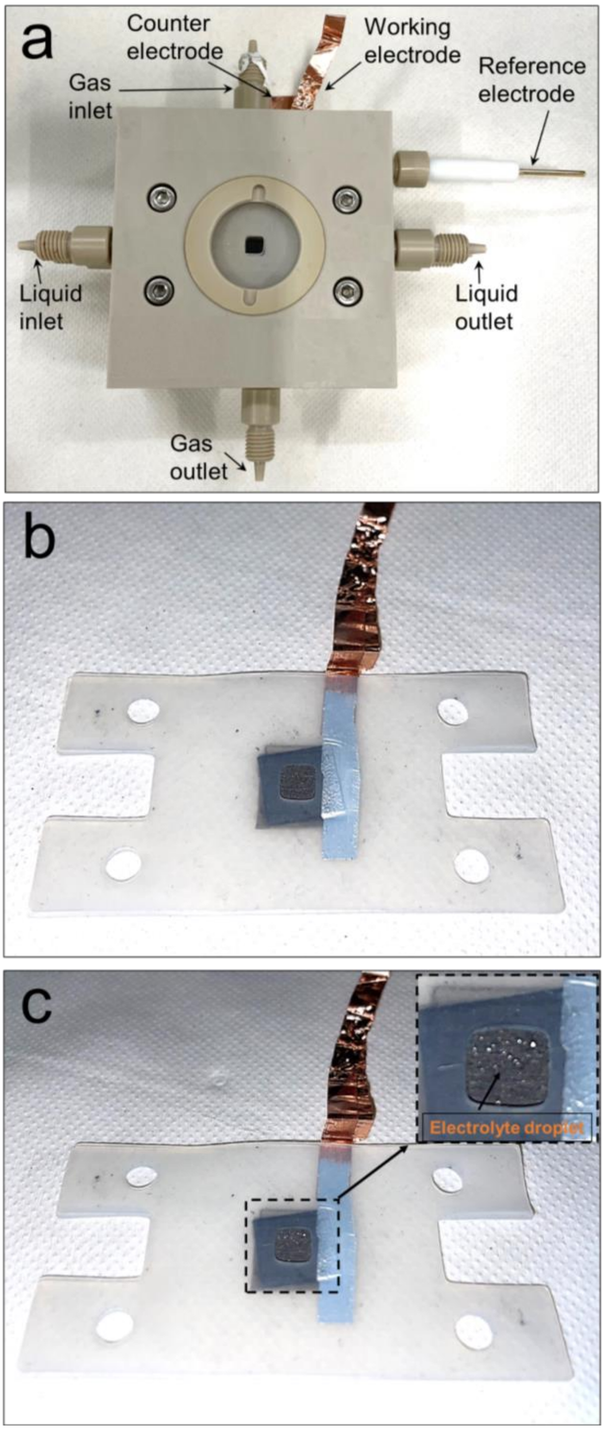

Figure S19. Flow cell set-up. (a) The digital picture of the flow cell set-up. (b) The digital picture of initial CoNCB@Gas diffusion electrode fixed with silicon membrane. (c) The digital picture of spent CoNCB@Gas diffusion electrode fixed with silicon membrane. The leaching electrolyte droplet would happen after long-time running, which would inhibit the oxygen transfer and decrease the active catalyst areas.

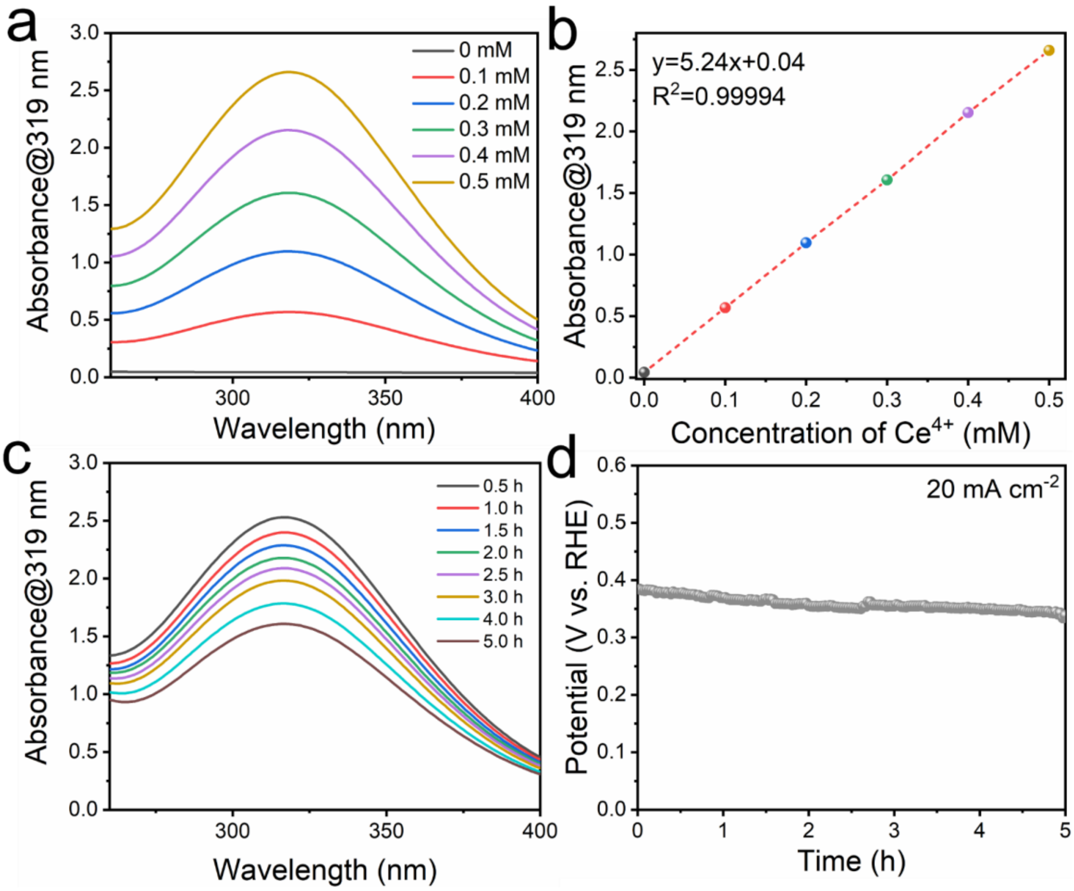

Figure S20. UV-vis titration test. (a) UV-vis absorbance spectra of standard $Ce(SO_4)_2$ solutions (up to 0.5 mM) in 0.5 M H_2SO_4. (b) The linear calibration curve (shown as an inset) at the peak wavelength (319 nm). (c) UV-vis absorbance spectra of Ce^{4+} solutions after reacting with electrolytes at different times. (d) Measured working potential with time at the fixed current density of 20 mA cm^{-2}.

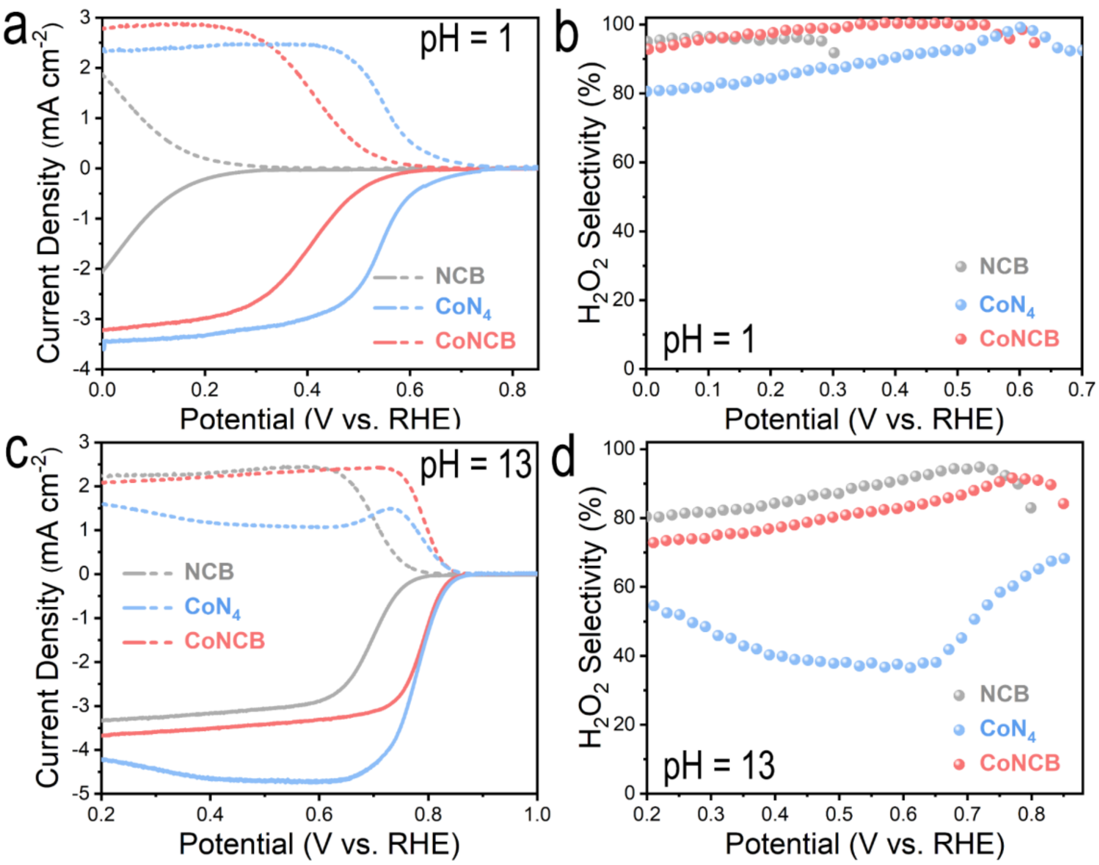

Figure S21.Effct of pH. (a, c) ORR polarization curves of disk current density (solid line) and ring current density (dash line) of NCB, CoN_4, and CoNCB in 0.1 M $HClO_4$ (pH = 1) and 0.1 M KOH (pH = 13). (b, d) Calculated H_2O_2 selectivity (H_2O_2 %).

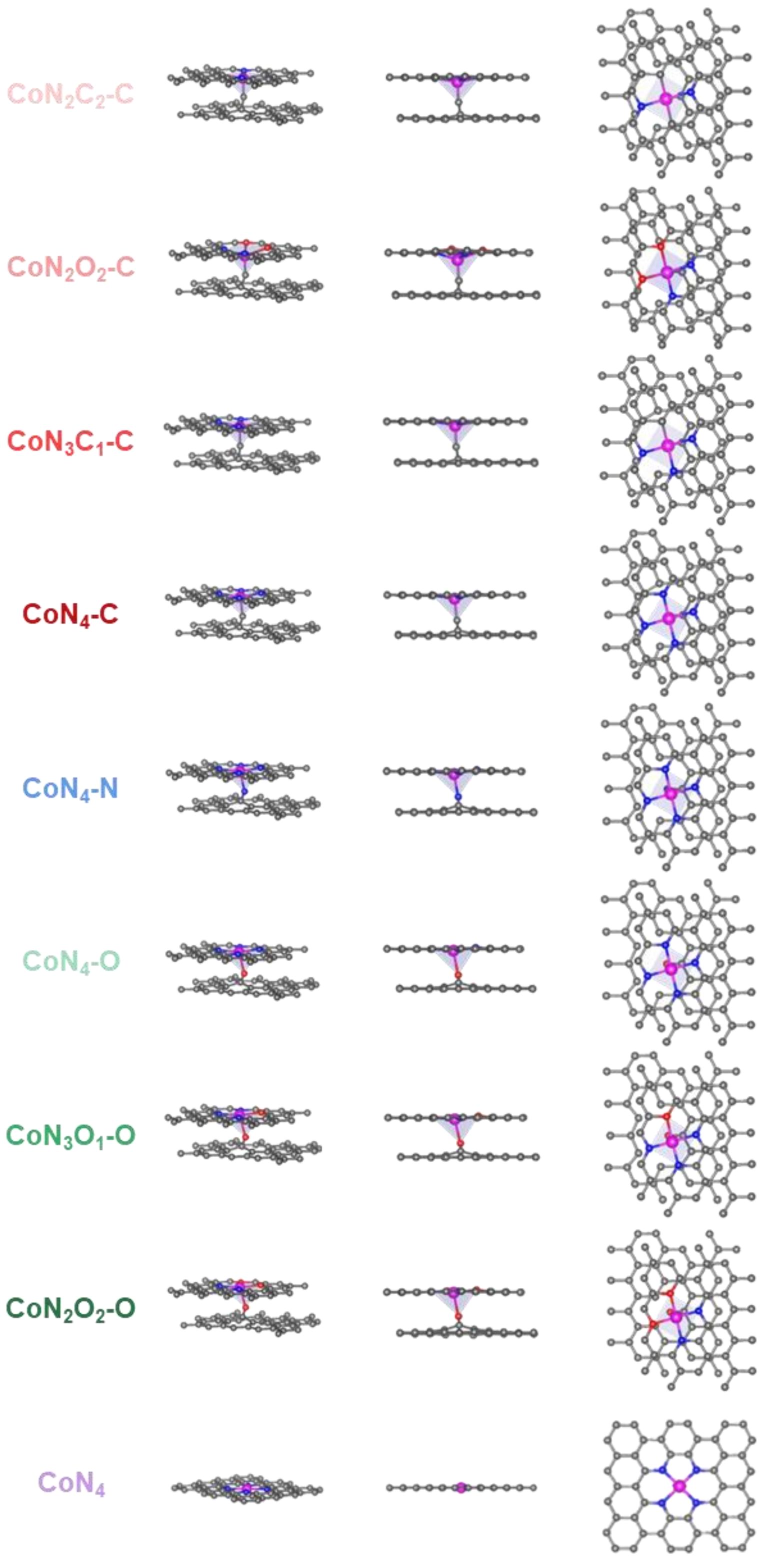

Figure S22. Different views of configurations including: CoN_2C_2-C, CoN_2O_2-C, CoN_3C_1-C, CoN_4-C, CoN_4-N, CoN_4-O, CoN_3O_1-O, CoN_2O_2-O, CoN_4. From left to right: main view, front view, and top view. Cobalt atoms are presented as purple, nitrogen as blue, oxygen as red, carbon as grey.

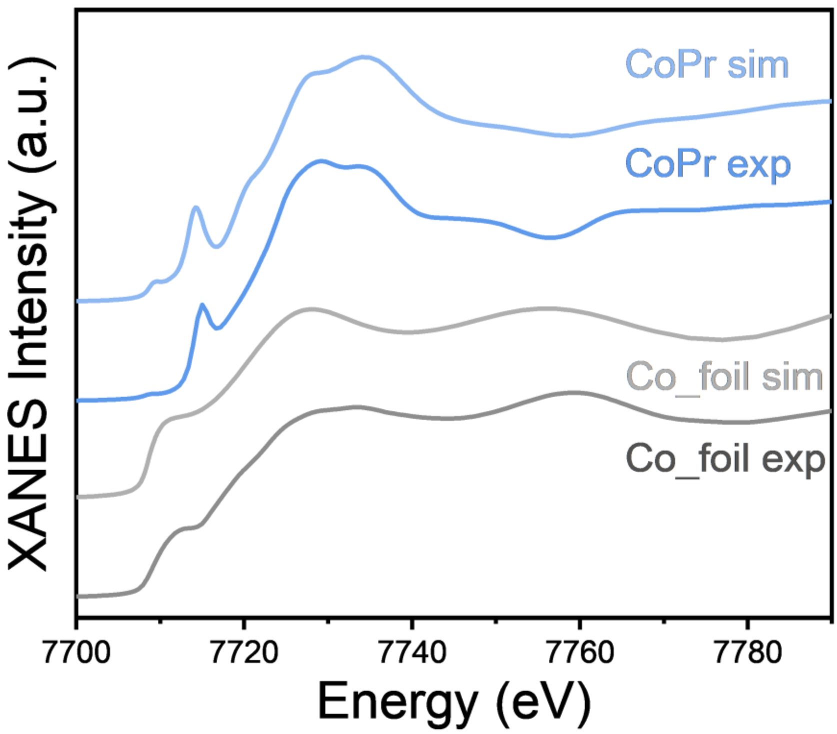

Figure S23. Experimental and simulated XANES spectra of Co foil and CoPr. The simulated (sim) XANES spectra of Co foil and CoPr reference aligned well with the experimental (exp) spectra.

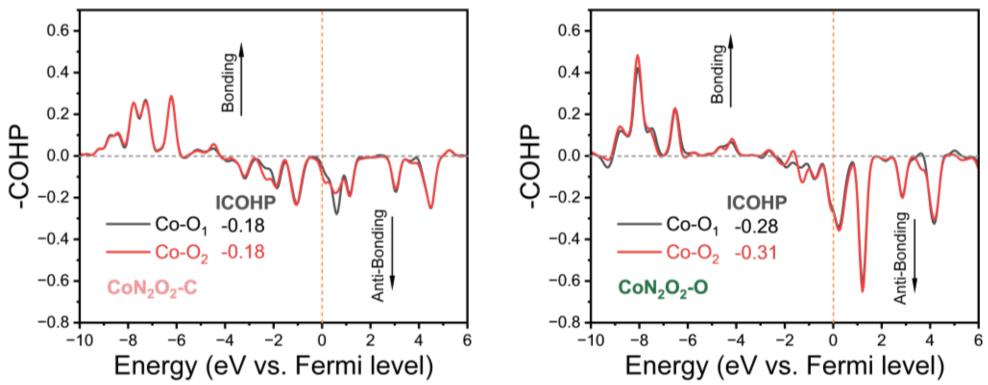

Figure S24. Projected Crystal Orbital Hamilton Population (COHP) curves of the interactions between Co atom and O atom in CoN_2O_2-C and CoN_2O_2-O configurations. This was performed using the Local Orbital Basis Suite Toward Electronic Structure Reconstruction (LOBSTER) package.

Supplementary Note 2. The reason that CoN_2O_2-C configuration with the largest off-center distance is an outlier is probably because the in-plane O is saturated by surrounding C atoms, leading to the weakened interaction between Co and O ligand. Moreover, the interaction between Co and axial C ligand is very active, thus pulling the Co far away from the plane center compared to that of axial N and O ligand. In addition, crystal orbital Hamilton population (COHP) method was employed to compare the interaction of Co-O ligand in CoN_2O_2-C and CoN_2O_2-O configurations. Compared to CoN_2O_2-O configuration, the COHP curve of CoN_2O_2-C configuration displays stronger overlap of antibonding states under the Fermi level, thus exhibiting more weakened bond interaction between Co and O ligands. This was further verified by the qualitative integrated COHP (ICOHP) (as shown in the picture). The more negative ICOHP indicates higher strength of bond interaction.

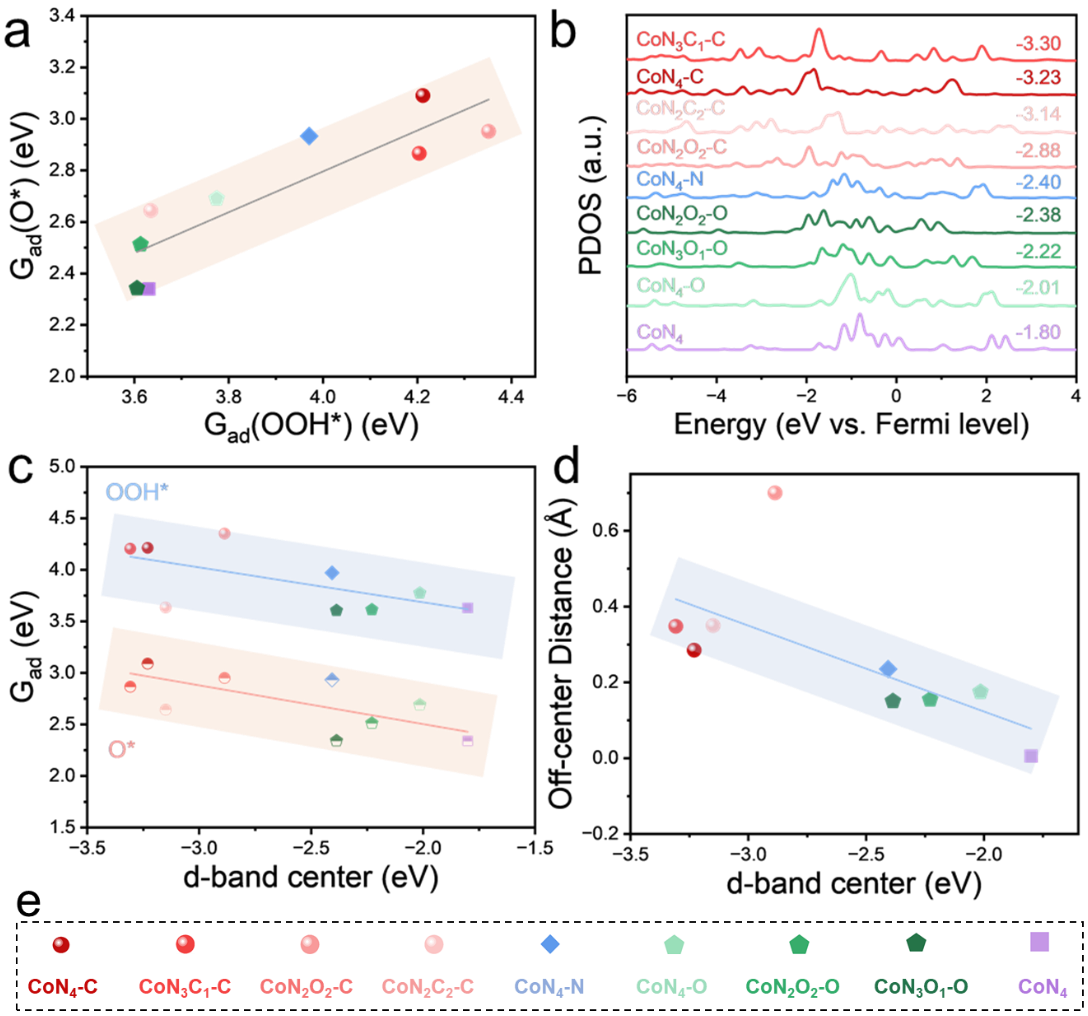

Figure S25. DFT calculations. (a) The relationship between calculated G_{ad}(OOH*) and G_{ad}(O*) of different models. (b) The projected density of states (PDOS) of different models. (c) The relationship between G_{ad}(OOH*), G_{ad}(O*) and d-band center of different models. (d) The relationship between off-center distance and d-band center of different models. (e) The symbols of different configurations.

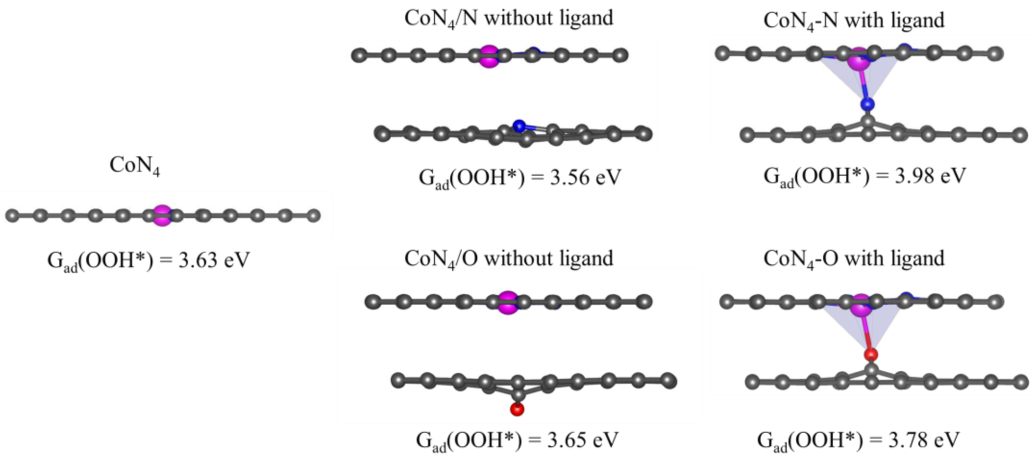

Figure S25. The models and corresponding adsorption energies without a carbon layer and a ligand (left), with a carbon layer but without a ligand (middle) and with both a carbon layer and a ligand (right).

Supplementary Note 3. While the impact of axial ligands in M-N-C configurations has been extensively studied, the role of an additional carbon layer has received limited attention.[1, 2] To evaluate the effect of additional carbon layer, we calculated CoN_4/N model and CoN_4/O model which incorporate CoN_4 with additional N- and O- doped carbon layer, respectively (**Figure S25**). A hydrogen atom was used for the saturation of the active N or O ligand. The G_{ad}(OOH*) of CoN_4/N model and CoN_4/O model is 3.56 eV and 3.65 eV, respectively, which is close to that of CoN_4 model (3.43 eV). In contrast, CoN_4-N and CoN_4-O with axial ligand, exhibit G_{ad}(OOH*) values of 3.98 eV and 3.78 eV, respectively, suggesting that axial ligand critically influences the G_{ad}(OOH*). Nevertheless, evaluating the role of the additional carbon layer in adjusting the bond length of the axial ligand and the off-center distance of the central Co atom is challenging. The model system is intricately balanced by coordinate bonds and Van der Waals interactions between the carbon layers. Therefore, we assume that the enhanced performance likely results from the synergetic effect of off-center distance, coordination atoms and length, and layer distance. While it is challenging to isolate the individual roles, we have provided this analysis in the supporting information, aiming to inspire future research in the field of single atom catalysts.

Table S5. The EHPP performance comparison of CoNCB and reported catalysts in neutral media.

Catalyst	Electrolyte	Potential @0.1 mA cm^{-2} (V)	Max. H_2O_2 Selectivity (%)	Potential @1 mA cm^{-2} (V)	Cobalt loading (wt %)	TOF (s^{-1})	Reference
CoNCB	0.1 M PBS (pH 7)	0.76	99.9	0.66	0.05	186	This work
O-CNT	0.1 M PBS (pH 7)	0.43	80	0.3	N.A.	N.A.	[3]
Fe-O-CNT	0.1 M PBS (pH 7.2)	0.48	90	0.42	N.A.	N.A.	[4]
O-C(Al)	0.1 M PBS (pH 7)	0.53	90	0.4	N.A.	N.A.	[5]
MCHS-9:1	0.1 M PBS (pH 8)	0.57	99	0.51	N.A.	N.A.	[6]
$OCNS_{900}$	0.1 M PBS (pH 7)	0.61	94	0.51	N.A.	N.A.	[7]
O-GOMC	0.1 M PBS (pH N.A.)	0.53	93	0.48	N.A.	N.A.	[8]
B-C	0.1 M Na_2SO_4 (pH N.A)	0.4	75	0.26	N.A.	N.A.	[9]
O-Co-N_2C_2	0.1 M PBS (pH 7)	0.79	99.8	0.72	0.66	11.57	[10]
Co-N SAC_{DP}	0.1 M PBS (pH 7.2)	0.71	88	N.A.	1.48	0.02	[11]
COF-366-Co	0.1 M PBS (pH 7.4)	0.42	96.	N.A.	0.78	0.04	[12]
Co-N2-C/HO	0.1 M PBS (pH 7.4)	0.58	90	0.35	0.88	0.35	[13]
Co_1-NG(O)	0.1 M PBS (pH 7.4)	0.58	68	0.4	1.4	1.65	[14]

Supplementary References

1. Zhao QL, *et al.* Approaching a high-rate and sustainable production of hydrogen peroxide: oxygen reduction on Co-N-C single-atom electrocatalysts in simulated seawater. *Energy Environ. Sci.* **14**, 5444-5456 (2021).

2. Pan Y, *et al.* Design of single-atom Co–N_5 catalytic site: a robust electrocatalyst for CO_2 reduction with nearly 100% CO selectivity and remarkable stability. *J. Am. Chem. Soc.* **140**, 4218-4221 (2018).

3. Lu ZY, *et al.* High-efficiency oxygen reduction to hydrogen peroxide catalysed by oxidized carbon materials. *Nat. Catal.* **1**, 156-162 (2018).

4. Jiang K, *et al.* Highly selective oxygen reduction to hydrogen peroxide on transition metal single atom coordination. *Nat. Commun.* **10**, 3997 (2019).

5. Yang Q, *et al.* Atomically dispersed Lewis acid sites boost 2-electron oxygen reduction activity of carbon-based catalysts. *Nat. Commun.* **11**, 5478 (2020).

6. Pang YY, Wang K, Xie H, Sun Y, Titirici MM, Chai GL. Mesoporous carbon hollow spheres as efficient electrocatalysts for oxygen reduction to hydrogen peroxide in neutral electrolytes. *ACS Catal.* **10**, 7434-7442 (2020).

7. Chen S, *et al.* Chemical identification of catalytically active sites on oxygen-doped carbon nanosheet to decipher the high activity for electro-synthesis hydrogen peroxide. *Angew. Chem. Int. Ed.* **60**, 16607-16614 (2021).

8. Lim JS, *et al.* Designing highly active nanoporous carbon H_2O_2 production electrocatalysts through active site identification. *Chem* **7**, 3114-3130 (2021).

9. Xia Y, *et al.* Highly active and selective oxygen reduction to H_2O_2 on boron-doped carbon for high production rates. *Nat. Commun.* **12**, 4225 (2021).

10. Shen H, *et al.* Boosting oxygen reduction for high-efficiency H_2O_2 electrosynthesis on oxygen-coordinated Co-N-C catalysts. *Small* **18**, e2200730 (2022).

11. Chen S, *et al.* Identification of the highly active Co-N_4 coordination motif for selective oxygen reduction to hydrogen peroxide. *J. Am. Chem. Soc.* **144**, 14505-14516 (2022).

12. Liu C, *et al.* Intrinsic activity of metal centers in metal-nitrogen-carbon single-atom catalysts for hydrogen peroxide synthesis. *J. Am. Chem. Soc.* **142**, 21861-21871 (2020).

13. Gong HS, *et al.* Low-coordinated Co-N-C on oxygenated graphene for efficient electrocatalytic H_2O_2 production. *Adv. Funct. Mater.* **32**, 2106886 (2022).

14. Jung E, *et al.* Atomic-level tuning of Co–N–C catalyst for high-performance electrochemical H_2O_2 production. *Nat. Mater.* **19**, 436-442 (2020).
